# Multiple Exciton Generation in 3D-Ordered Networks of Ge Quantum Wires in Alumina Matrix

**DOI:** 10.3390/ma15155353

**Published:** 2022-08-03

**Authors:** Marija Tkalčević, Denis Boršćak, Ivana Periša, Iva Bogdanović-Radović, Iva Šarić Janković, Mladen Petravić, Sigrid Bernstorff, Maja Mičetić

**Affiliations:** 1 Ruđer Bošković Institute, Bijenička cesta 54, 10000 Zagreb, Croatia; marija.tkalcevic@irb.hr (M.T.); denis.borscak@irb.hr (D.B.); ivana.perisa@irb.hr (I.P.); iva@irb.hr (I.B.-R.); 2 Faculty of Physics and Center for Micro- and Nanosciences and Technologies, University of Rijeka, Radmile Matejčić 2, 51000 Rijeka, Croatia; iva.saric@phy.uniri.hr (I.Š.J.); mpetravic@phy.uniri.hr (M.P.); 3 Elettra Sincrotrone, S.C.p.A., Strada Statale 14, km 163.5 in AREA Science Park, Basovizza, 34149 Trieste, Italy; sbernstorff@elettra.eu

**Keywords:** Ge quantum wires, multiple exciton generation, quantum efficiency, thin films, sensors, solar cells

## Abstract

Thin films containing 3D-ordered semiconductor quantum wires offer a great tool to improve the properties of photosensitive devices. In the present work, we investigate the photo-generated current in thin films consisting of an interconnected 3D-ordered network of Ge quantum wires in an alumina matrix. The films are prepared using nitrogen-assisted magnetron sputtering co-deposition of Ge and Al_2_O_3_. We demonstrate a strong photocurrent generation in the films, much stronger than in similar films containing Ge quantum dots. The enhanced photocurrent generation is the consequence of the multiple exciton generation and the films’ specific structure that allows for efficient carrier transport. Thin film with the largest nitrogen content showed enhanced performance compared to other thin films with 1.6 excitons created after absorption of a single photon at an energy nearly equal to the double bandgap value. The bandgap value depends on the geometrical properties of the quantum wires, and it is close to the maximum of the solar irradiance in this case. In addition, we show that the multiple exciton generation is the most pronounced at the photon energy values equal to multiple values of the thin film bandgap.

## 1. Introduction

Numerous types of photoelectric conversion materials have been investigated in the past decade in order to increase efficiency and reduce the costs of photosensitive devices. The properties of low-dimensional semiconductor nanostructures can easily be tuned by controlling the nanomaterial size, which allows for the manipulation of the structural properties [1]. This makes them excellent candidates for enhancing energy conversion efficiencies and boosting the devices’ performance [1,2,3,4,5,6,7].

A primary advantage of germanium-based technology is its compatibility with existing silicon processes. It also has many excellent properties usable for different applications, especially in photodetectors and photovoltaics. Germanium is a pseudo-direct bandgap material because of the small energy difference (0.136 eV) between its direct and indirect bandgap. The bandgap of bulk germanium is around 0.67 eV. Germanium shows a very strong quantum confinement effect due to the large Bohr exciton radius of germanium (24 nm), which is much larger than the Bohr exciton radius of silicon (5 nm). Therefore, it is easier to exploit these properties for practical purposes. Ge QDs have been successfully employed to increase a Si-based photodetector’s quantum efficiency (QE) [1]. When embedded in dielectric matrices, Ge QDs often exhibit very strong confinement effects. The bandgap tuning is easily achieved for an extensive energy range from 0.7 up to 3.2 eV [3,4]. Another way to modify the bandgap is by applying tensile strain to Ge nanostructures. Tensile strain can make germanium more direct bandgap-like because it shrinks the direct bandgap more than the indirect bandgap due to the difference in deformation potentials of the direct Γ valley and the indirect L valleys [8]. The quantum confinement effect can be counteracted by applying tensile strain in Ge NWs [9]. Carrier mobility is considerably larger in germanium compared to silicon, and since it has a smaller bandgap compared to silicon, it is expected to perform at longer wavelengths extending well into the NIR region of the spectrum [10]. Ge NWs and Ge QDs have been successfully implemented for enhancement performances of photovoltaic devices [9,11,12,13].

Multiple exciton generation (MEG) is another mechanism that could improve the efficiency of photosensitive devices. Multi-exciton generation is a process in which two or more electron–hole pairs after the absorption of a single photon. By way of simple energy conservation arguments, the minimal amount of energy required to produce a bi-exciton is twice the energy of the quantized bandgap [3]. The efficiency of MEG states is mainly influenced by a kinetic competition between the MEG process and ultra-fast hot carrier cooling of the photo-generated state. This process is much more likely to occur in nanostructured semiconductor materials than in bulk because of the quantum confinement effect. Imperfections on the surface of quantum dots lead to less than ideal efficiencies for MEG in semiconductor nanostructures. Their number is significantly enhanced at the semiconductor/high-k oxide dielectric interfaces like Al_2_O_3_ [14]. Trapped charge carriers reduce the ability of quantum dots to undergo MEG. Photo-generated charge carriers may either reside in Coulomb-bound excitons, or they may exist as free charges; only in the latter case do the charge carriers contribute to photoconductivity. Sandeep, C. S., et al. demonstrated that, for mobilities above 1 cm^2^ V^−1^ s^−1^, electrons and holes separate rapidly enough to escape Auger recombination [15].

Multiple exciton generation has been reported for germanium quantum dots in Refs. [4,5,16]. In most of these cases, the Ge-based materials were annealed in a nitrogen atmosphere or nitrogen was present during the deposition. Annealing in nitrogen significantly reduces the number of defects at the semiconductor/high-k dielectrics [14,17]. Therefore, it is widely used to increase the efficiency of photosensitive devices [14]. Nitrogen doping has been investigated extensively in silicon, but there is limited information on its interaction with vacancies in germanium, despite most point defect processes in germanium being vacancy controlled. Kuganathan N. et al. showed that the N doping process introduces gap states, which lead to bandgap narrowing [18].

Our previous work demonstrates weak MEG in Ge QDs in alumina matrix, prepared in nitrogen-rich working gas during magnetron sputtering deposition [19]. The present paper investigates the photoelectric conversion properties and MEG probability in Ge QWs embedded in the amorphous alumina matrix. The material consists of 3D mesh of the inter-connected Ge QWs. So, the Ge nano-objects have significantly different shape with respect to the already investigated Ge QDs, and their interconnectivity. We demonstrate that the MEG happens also in the Ge QWs due to the nitrogen addition. The effect is significantly stronger than the one observed in films with Ge QDs. In contrast to the case of Ge QDs in an alumina matrix, multiple excitons are generated in Ge QWs already in the as-grown films; thus, no additional annealing is needed. Finally, we show that a much stronger photo-generated current is obtained in the films with Ge QWs compared to samples with Ge QDs. This is because Ge QWs are interconnected through the entire thin film, allowing for more efficient charge transport than in the isolated QDs. We believe that the above advantages of Ge QWs films open the possibility for their application in solar cells, sensors, photodetectors, and other photosensitive devices.

## 2. Materials and Methods

### 2.1. Sample Preparation

Magnetron sputtering deposition is a thin film growth technique that allows for precise control over the deposition parameters. Thin films containing Ge nanowire networks were prepared by simultaneous co-deposition of Ge and Al_2_O_3_ using a magnetron sputtering KJLC CMS-18 system on Si (100) and quartz substrates for 30 min. The structure of the films is expected to be the same on both substrates. The films deposited on the Si substrate are used for electrical measurements and the quartz substrate for optical. We used 3-inch Ge (99.999%) and Al_2_O_3_ (99.999%) targets produced by K.J. Lesker.

Before deposition, the substrates were cleaned in an ultrasonic bath with acetone, ethanol, and isopropanol for 5 min to remove any dirt and grease. Before loading in the deposition chamber, the substrates were cleaned with deionized water and dried in nitrogen gas. Argon is used as the working gas, and nitrogen was added in different amounts, as indicated in Table 1. The total pressure (sum of partial pressures of Ar and N_2_) was kept at the constant value of 3.5 mTorr, during all depositions. The base pressure in the chamber prior to the deposition was 10^−8^ mTorr. Nitrogen enters the chamber at the place close to the substrate. The deposition conditions, including the sputtering powers for Ge and Al_2_O_3_, the deposition temperature, and the nitrogen flow rate, are given in Table 1. After the deposition, the films consisted of a 3D mesh of Ge nanowires embedded in an Al_2_O_3_ matrix. After deposition, samples were additionally annealed at 600 °C for 40 min under a high vacuum.

**Figure 1:**
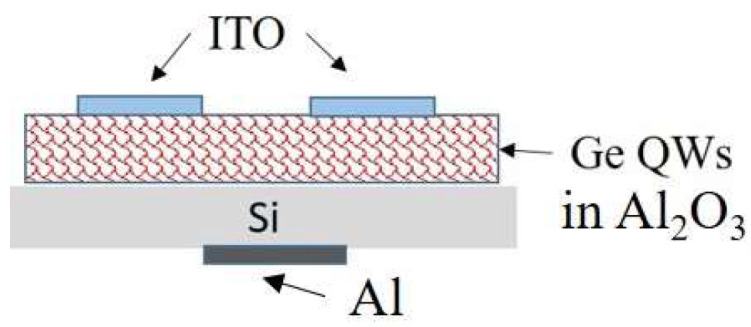
Scheme of the device for the *I-V* and QE measurements.

For the determination of the photo-electrical properties, electrodes were added to the films using magnetron sputtering deposition to obtain a heterojunction photodiode with the structure indium-tin-oxide (ITO)/Al_2_O_3_ + Ge/Si/Al, as shown in Figure 1. The ITO electrodes have a thickness of about 50 nm and an area of 9 mm^2^. Al electrode has an area of 1 cm^2^ and a thickness of 100 nm.

### 2.2. Sample Characterization

A powerful method for the statistical structure analysis (QW shape, size, and ordering properties) is Grazing Incidence Small Angle X-ray Scattering (GISAXS), while Grazing Incidence Wide Angle X-ray Scattering (GIWAXS) can be used for the crystalline structure determination. We employed these techniques for the structural characterization at the SAXS beamline of the synchrotron Elettra (Trieste, Italy) using a photon energy of 8 keV. The scattered radiation was collected simultaneously by a two-dimensional Pilatus3 1M detector (GISAXS) and a 2D Pilatus 100k detector (GIWAXS). The grazing incidence angle was slightly above the critical angle of total reflection.

The optical properties (transmission and reflection) of the prepared films were examined by Ocean Optics equipment, including a deuterium-halogen light source (DH–2000 –BAL) and a UV/VIS detector (HR 4000) and the SpectraSuite software, version 2.0.

The elemental composition of the samples was measured by Time-of-Fight Elastic Recoil Detection Analysis (TOF-ERDA). The measurements were done with a 20 MeV beam of ^127^I^6+^ ions at an impact angle of 20° between the sample surface and the beam and 37.5° between the spectrometer and the beam.

The oxidation states of Ge were obtained from X-ray photoemission spectroscopy (XPS) measurements in a SPECS XPS instrument equipped with a hemispherical electron analyzer and monochromatized Al Kα X-rays of 1486.74 eV. The photoemission spectra were taken around the Ge 2p core levels and simulated with mixed Gaussian–Lorentzian (G-L) functions with Shirley background subtraction [20]. The binding energy scale was calibrated against the position of the C 1s peak placed at 284.5 eV.

For the current-voltage (*I-V*) and quantum efficiency (QE) measurements, we used a Photovoltaic Testing System–Spectral Response and Quantum Efficiency from Sciencetech (White Light Quantum Efficiency System), in the spectral range from 320 nm to 1200 nm and under a bias voltage of 5 V. All *I-V* measurements were done in the dark and under illumination to test the photoresponse. The current-voltage characteristics were measured in the range from −4 V to 3V. The *I-V* measurements were taken upon illumination of the samples with one sun (100 mW/cm^2^), obtained by a 150 W Xenon lamp and an AM 1.5 G filter.

## 3. Results

### 3.1. Structural Properties

#### Ge QWs Network Properties

**Figure 2:**
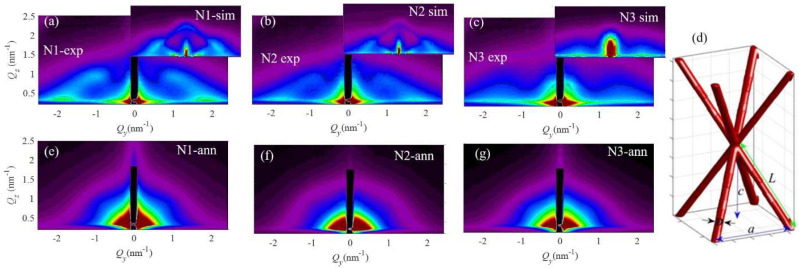
(**a**–**c**) GISAXS maps, and their simulations in the insets, of the as-grown films. (**d**) Model of the QW network and its main parameters. (**e**–**g**) GISAXS maps of the annealed films, showing the destruction of the QW regular network that was present before annealing.

The shape and arrangement properties of the quantum structures formed in the films N1-N3 were determined from the GISAXS maps shown in Figure 2. The GISAXS maps of the as-grown films are shown in Figure 2a–c. They reveal the formation of an ordered Ge QW network, as explained in detail in Ref. [21]. The scheme of the formed QWs and their ordering in a QW network is illustrated in Figure 2d. The nodes of the QW network are ordered in a body-centered tetragonal lattice, and the QWs are interconnected. The interconnection is well visible in the microscopy measurements published in Ref. [21] and additionally confirmed by Ge desorption from this type of materials as shown in Ref. [22]. The side peaks are visible in the maps of Figure 2a–c for all as-deposited samples, with the center at *Q*_y_ of about 2 nm^−1^. The similar position of the center for all side peaks indicates a similar separation of all QWs formed in the plane parallel to the substrate. The films N1 and N2 show additional Bragg spots centered at the position of (*Q*_y_, *Q*_z_) = (1, 1) nm^−1^. These peaks correspond to the formation of the ordered QW network, as reported in [21]. The peak positions are similar, showing that the geometry of the formed QW networks is similar. The peaks are most intense for the film without nitrogen (N1). They are weaker for the film N2 and absent for the film N3, indicating an increase of disorder in the QW positions with the addition of N. Thus, we can conclude that all samples have a similar structure, but the ordering quality of the QWs is the lowest for the highest nitrogen concentration.

We also performed a numerical analysis of the GISAXS maps of the films N1, N2, and N3 to obtain additional details for the QWs size and ordering properties. We assumed the formation of a QWs network with the body-centered tetragonal ordering of the network nodes, as shown in Figure 2d. The position of the nodes deviates from the ideal ones following the paracrystal model, as described in [21,23]. The parameters obtained from the fit of the GISAXS maps are given in Table 2. From these parameters, it is evident that the QWs have a diameter (parameter *D*) of about 1.1 nm, length (parameter *L*) of about 2.9 nm, and lateral separation (parameter *a*) of about 4 nm. The vertical separation of the QW nodes (parameter *c*) is approximately 3.5 nm, increasing slightly with the nitrogen concentration. The deviation parameters ($\sigma_{1,2}^{x,y},\;\sigma_{1,2}^{z},\;\sigma_{3}^{x,y},\;\sigma_{3}^{z}$) are slightly increasing with the nitrogen concentration. The film thickness (*d*) is also very slightly increasing with N content.

The measured GISAXS maps of the annealed films (Figure 2e–g) display no presence of the Bragg spots, which are visible for the as-grown films. The absence of Bragg spots indicates the decomposition of the ordered QWs structures present before the annealing. After annealing, the shape of the QWs changed, so they are much closer to a sphere. This follows from the semi-circular shape of the GISAXS intensity signal, originating from nearly spherical nano-objects. A similar, less pronounced effect was observed for elongated Ge QDs in Ref. [21]. We argue that a lattice of non-ordered Ge QDs is formed after annealing. In comparison, the annealing of Ge QDs in a high vacuum (700 °C, 45 min) did not change their shape properties [19].

**Figure 3:**
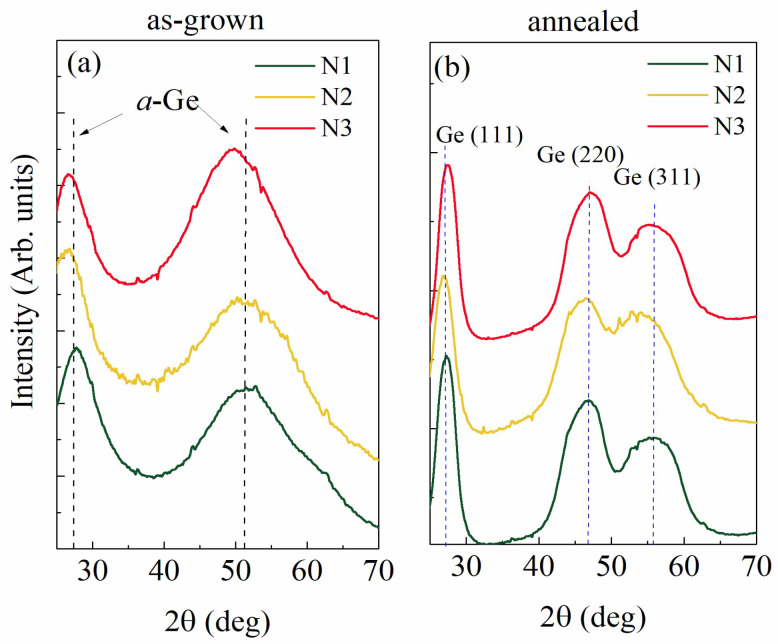
Grazing incidence wide-angle X-ray scattering (GIWAXS) of the (**a**) as-grown and (**b**) annealed films. The position of the amorphous Ge (a-Ge) and crystalline Ge (c-Ge) peaks are denoted by dashed lines.

The crystalline structure of the films was determined by wide-angle X-ray scattering (GIWAXS). The measured data for all films are presented in Figure 3. The as-grown films, shown in Figure 3a, are fully amorphous, and only the peaks related to amorphous Ge (a-Ge) are visible. The annealing leads to the crystallization of Ge, as follows from the measurements shown in Figure 3b. The crystalline Ge (111), (220), and (311) peaks are visible for all films. The width of the peaks is similar for all annealed films implying that the Ge crystallite sizes are similar for all films. The size of the Ge crystallites of 1.2 ± 0.2 nm for all films was estimated from the Debye–Scherrer formula.

### 3.2. Atomic Composition and Ge Oxidation

The atomic composition of the films, measured by the TOF-ERDA technique, is given in Table 3. The aim of these measurements was to determine the atomic concentration of nitrogen incorporated in the thin films, during the deposition of films. Before annealing, the film N1 has a very small amount of N (0.6%), in agreement with the absence of N during the deposition. However, the films N2 and N3 have 2.4 and 3.6 atomic percentages of nitrogen, respectively, reflecting the incorporation of N into the material during the deposition. The corresponding N concentrations in the annealed films are similar to those before annealing. The total number of Ge atoms is 190 at/cm^2^, 188 at/cm^2^, and 194 at/cm^2^ for the as-grown films N1, N2, and N3, respectively.

GISAXS measurements have confirmed that the initial regular ordering of QWs was destroyed during annealing and that nearly spherical QDs were formed instead. The atomic composition analysis revealed that the atomic percentages change only slightly, within a few percent for all films, both as-grown and annealed. It is consistent with a proposition that the Ge atoms only rearrange their positions during the annealing, thus changing the shape of the formed structure from quantum wires to spherical quantum dots.

The oxidation of semiconductor surfaces has been extensively studied during the last decade because of its vast technological interest. Germanium is an attractive candidate for manufacturing complementary metal-oxide-semiconductor (CMOS) elements and metal-insulator-semiconductor devices in large-scale integrated circuits [24]. Since Ge QDs are prone to oxidation, the role of the interface cannot be ignored. The oxidation problem is even more complex when Ge QDs are embedded in oxide-rich matrices, such as Al_2_O_3_ or SiO_2_. Ge QDs in SiO_2_ fabricated by magnetron sputtering have an interface shell with Ge-oxide states, resulting in a moderate bandgap tuning [25]. Oxide-rich areas significantly impact the optical absorption in Ge QDs shown in [26]. In addition, it was demonstrated that Al reacts with oxygen and can even pull the oxygen out from GeO_2_. Ge-Ge and Ge-Al bonding units are then formed at the Al/GeO_2_ interface, which influences the photocurrent [27].

**Figure 4:**
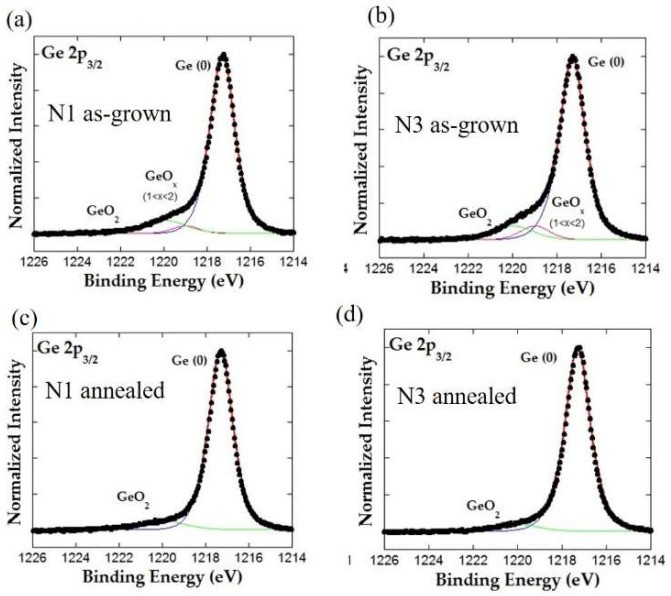
XPS measurements for the (**a**,**b**) as-grown and (**c**,**d**) annealed samples N1 and N3. The Ge oxidation states are indicated in the figure.

The chemical state of Ge can be determined from XPS analysis around Ge 2p core levels. The large spin-orbit splitting of Ge 2p_3/2_ and 2p_1/2_ core levels of about 31 eV enables the photoemission studies around only one component, Ge 2p_3/2_. In addition, chemical shifts between elemental Ge and Ge oxides, GeO and GeO_2_, of around 1 and 3 eV, respectively, are easily resolved in the high-resolution XPS measurements. The results of the XPS measurements of the as-grown and annealed films, N1 and N3, respectively, are shown in Figure 4. The surfaces of all films are firstly sputter-cleaned within the analytical UHV chamber by a low-energy argon ion-beam bombardment to remove the surface contamination. All photoemission peaks display similar shapes indicating that nitrogen does not significantly influence the oxidation rate of Ge. The data are fitted with sets of G-L functions to distinguish the contribution of the elemental, metallic Ge (denoted by Ge (0)) from the contribution from Ge oxides (GeO_2_ and GeO_x_). The contribution of metallic Ge is dominant for all measured films.

The atomic percentage of particular states of Ge, as determined from XPS, is given in Table 4. The XPS analysis was repeated several times at different probing depths, obtained by sputter-removal of the sample surface by low-energy Ar^+^ bombardment. The results for all probing depths were similar, except for the surface layer, showing the presence of significantly more Ge oxides.

### 3.3. Optical Properties

**Figure 5:**
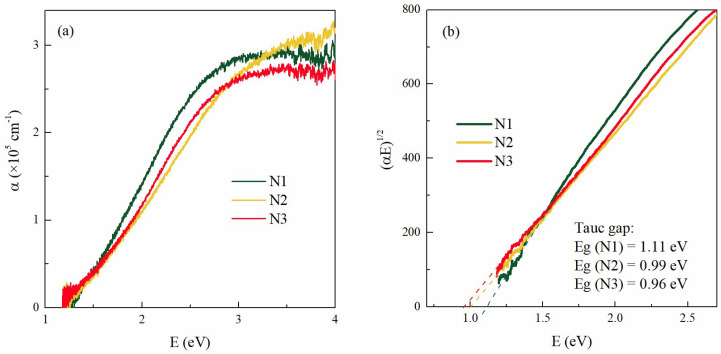
(**a**) The absorption coefficient of the films as a function of energy. (**b**) Tauc’s plot (αE)^1/2^ vs. energy was used to determine the bandgaps of thin films.

The measurements of the absorption coefficients of the as-grown films N1, N2, and N3 are summarized in Figure 5. The optical properties of annealed films were not considered as we were focused on the properties of QWs. The dependence of the absorption coefficients (α) on the photon energy for all as-grown films is shown in Figure 5a. The absorption is similar for all samples. The energy gap of each film was estimated from the Tauc plot, as shown in Figure 5b. A linear relationship was found for (αE)^1/2^ vs. energy plots, which is characteristic of indirect transitions between the allowed bands. The values of the energy gaps are given in Figure 5b. The highest bandgap value is found for film N1 (1.11 eV), with no nitrogen added and the smallest diameter, while the smallest bandgap is found for film N3 (0.96 eV), with the largest QW diameter. Thus, the value of the bandgap slightly decreases with the QW diameter increase, as is expected for germanium, which shows strong quantum confinement effects.

### 3.4. Electrical Properties and Photocurrent

**Figure 6:**
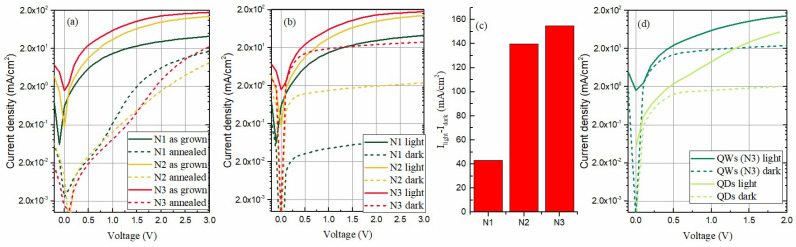
(**a**) *I-V* properties of the as-grown and annealed films under illumination, (**b**) I-V measurements of the as-grown films in the dark and under illumination, (**c**) difference of the current in the light and dark for the as-grown films, and (**d**) comparison of the *I-V* measurements for the QWs and QDs.

The current-voltage (*I-V*) properties of the films are shown in Figure 6. The photogenerated current properties of the as-grown and annealed films are compared in Figure 6a. It is obvious that the current values in as-grown films are much higher. This result can be explained with the strong interconnection of the Ge QWs in as-grown films, while the annealed films contain only isolated Ge QDs.

The *I-V* properties of the as-grown films in the dark and under illumination are compared in Figure 6b. We first note that the strongest photocurrent is generated in the as-grown film N3, containing the highest amount of nitrogen. The current is decreasing with the N amount for the other as-grown films.

Although the film N3 has the strongest current under illumination, it also has the strongest dark current. Both illumination and dark currents are decreasing with the decrease in the amount of nitrogen. The difference between the light and dark current is shown in Figure 6c. The difference is most significant for the film N3 deposited with the highest nitrogen content.

Finally, for comparison, the *I-V* properties of the films with QWs and the films with QDs prepared at 500 °C, as reported in [19], both deposited in the presence of nitrogen, and with the same type of contacts as reported in the Methods, are shown in Figure 6d. The current from the film with QWs is much stronger than the one from the film with QDs. The current values for the QDs are similar to those for the annealed samples shown in Figure 6a. As discussed above, the annealed films predominantly contain QDs, as explained earlier. The interconnection of the QWs plays an essential role in improving the photogenerated current.

In the case of QDs, the additional annealing improves the photocurrent [19], but the annealing has the opposite effect in the case of QWs, as evident from Figure 6a. We point out once again that annealing causes the formation of QDs from the QWs, and the interconnection of QWs is lost. Therefore, in addition to the nitrogen content, the interconnection of the Ge has an important role in photocurrent generation. A similar effect is reported and explained in Ref. [28]. Although the interconnection of Ge nano-objects reduces the confinement effects, it acts beneficially on the photo-current generation.

### 3.5. Quantum Efficiency Measurement

Spectral response measurements were used to estimate the efficiency of incident photon-electron conversion. The spectral response can represent the ability of each incident photon to be converted into electrons transported to the external circuit, called Quantum Efficiency (QE) [29].

**Figure 7:**
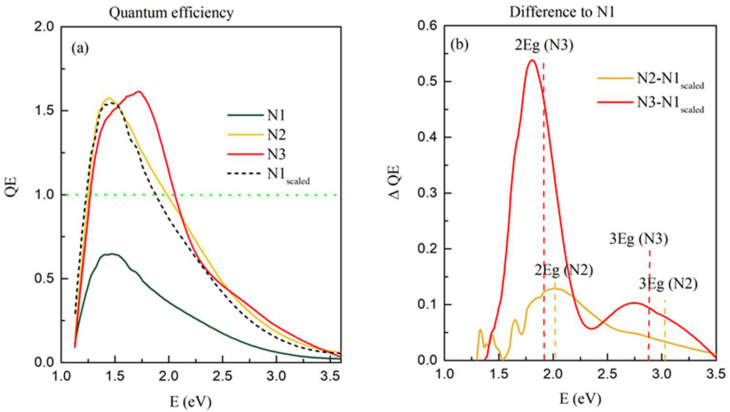
(**a**) Quantum efficiency of the films N1–N3. A green dashed line indicates the value of QE = 1, which corresponds to one exciton formed for one incident photon. N1_scaled_ represents the QE of the film N1 normalized to the maximum of the QE of the film N2. (**b**) Difference of the QE values measured for the film N2 and N3 with respect to the value of N1_scaled_.Vertical dashed lines indicate the positions of 2Eg and 3Eg for films N2 and N3.

Measurements of QE, i.e., the number of electrons produced by the total number of incident photons, of the as-grown films N1–N3, are shown in Figure 7a. An interesting feature is the intensity of the QE curves. For the thin films N2 and N3, the intensity of the QE curves is significantly higher than for N1. This means that a single photon forms more than one exciton, i.e., multiple exciton generation occurs in films N2 and N3. This fact also agrees with the strong photocurrent observed in films N2 and N3, shown in Figure 6a,b. Therefore, the multiple exciton generation occurs only in the films deposited with the presence of nitrogen.

As all films have a similar structure and absorption properties. We compared the QE curves’ shapes to examine the differences. For that purpose, the QE curve of N1 was normalized to get a similar maximum as the QE of the film N2 (denoted by N1_scaled_ in Figure 7a). The curves measured for the films N2 and N3 contain additional contributions compared to N1_scaled_. To analyze this effect in more detail, we subtracted N1_scaled_ from the curves measured for the films N2 and N3, where multiple exciton generation was observed. The resulting curves are shown in Figure 7b. Both subtracted curves show two clear peaks with similar maxima positions at values close to two or three times the value of their corresponding bandgaps Eg (see Figure 5 for the bandgap values). The higher N percentage in the films causes stronger multiple exciton generation effects. The N decreases the number of fixed oxide charges in high-k dielectrics such as the Al_2_O_3_ matrix [14,17]. The oxide charges occur primarily at the oxide/semiconductor interface, which is Ge QW/Al_2_O_3_ in our case. Due to the nano-size of the formed Ge QW mesh, this interface area is vast, as well as the number of these defects. Nitrogen reduces their number significantly, so the result of its addition is significantly reduced recombination of the photo-created carriers in the films with higher nitrogen content. In addition, the threshold energy for MEG is sensitive to surface defects; and nitrogen may reduce it [3].

## 4. Conclusions

In conclusion, we investigated the photo-generated current in thin films consisting of a 3D-ordered network of Ge quantum wires in an alumina matrix prepared by magnetron sputtering deposition in a nitrogen environment. The measured photo-generated current in all films is much stronger than the current previously measured from similar films of Ge QDs in the same matrix. The first reason for the strong photocurrent is the pronounced multiple exciton generation, which occurs in the films deposited with the presence of nitrogen. The nitrogen decreases the number of defects at the quantum wire/matrix interface and possibly also reduces the threshold energy for multiple exciton generation. The second important factor is the interconnection of the 3D mesh of Ge QWs, which ensures efficient transport of the charge carriers. We believe the presented materials are up-and-coming for application in various photosensitive devices.

## Figures and Tables

**Table 1:** Deposition parameters of the produced Ge QWs in the Al_2_O_3_ matrix. F_N_—N_2_ flow rate, *T*—deposition temperature, and *P*—sputtering power of the target.

Sample Name	*P* (Al_2_O_3_)/W	*P* (Ge)/W	*T*/℃	F_N_/sccm
N1	130	10	500	0
N2	130	10	500	2
N3	130	10	500	3

**Table 2:** Parameters of the formed QW lattices of the as-grown films found by GISAXS analysis: in-layer dot separation *a*, multilayer period *c*, deviations of the QW positions from the ideal ones $(\sigma_{1,2}^{x,y},\;\sigma_{1,2}^{z},\;\sigma_{3}^{x,y},\;\sigma_{3}^{z}$), QW diameter (*D*) and length (*L*), the standard deviation of their distribution $\sigma_{R},$ and the film thicknesses *d*. All values are given in nm.

Sample Name	*a*	*c*	$\sigma_{1,2}^{x,y}$	$\sigma_{1,2}^{z}$	$\sigma_{3}^{x,y}\;$	$\sigma_{3}^{z}\;$	*D*	*L*	$\sigma_{R}\;$	*d*
N1	4.1	3.6	1.3	0.68	0.67	0.1	1.08	2.8	0.33	51
N2	4.3	3.7	1.4	1.2	0.97	0.56	1.14	2.9	0.37	54
N3	3.9	3.9	1.5	1.2	---	---	1.04	2.9	0.33	59

**Table 3:** The atomic composition of the films was measured by TOF-ERDA. The values for the as-grown films are denoted by ‘ag’ and for the annealed films by ‘an’. All values are given in atomic percentages (at %).

Sample Name	N ag	Ge ag	O ag	Al ag	N an	Ge an	O al	Al an
N1	0.6 ± 0.1	55 ± 3	28 ± 1	8.7 ± 0.8	0.3 ± 0.1	50 ± 3	30 ± 1	13.4 ± 10
N2	2.4 ± 03	54 ± 3	28 ± 1	1.5 ± 0.2	1.5 ± 0.2	44 ± 2	34 ± 2	11.0 ± 0.8
N3	3.6 ± 0.4	47 ± 2	34 ± 2	3.8 ± 0.4	3.8 ± 0.4	44 ± 2	32 ± 2	4.8 ± 1

**Table 4:** Ge oxidation states are determined by XPS analysis. All values are given in atomic percent (at %).

Sample Name	Ge (0)	GeO_2_	GeOx
N1 ag	92.6	7.4	0.0
N1 an	84.5	6.5	6.5
N3 ag	86.0	10.4	3.6
N3 an	94.9	5.1	0.0

## Data Availability

Mičetić, Maja (2022), “Multiple Exciton Generation in 3D-Ordered Networks of Ge Quantum Wires in Alumina Matrix”, Mendeley Data, V1, doi:10.17632/gc34s8yjnz.1.
